# Ligand-specific conformational transitions and intracellular transport are required for atypical chemokine receptor 3–mediated chemokine scavenging

**DOI:** 10.1074/jbc.M117.814947

**Published:** 2017-11-27

**Authors:** Nicolas Montpas, Geneviève St-Onge, Nassr Nama, David Rhainds, Besma Benredjem, Mélanie Girard, Gilles Hickson, Véronique Pons, Nikolaus Heveker

**Affiliations:** From the ‡ Department of Biochemistry and Molecular Medicine, University of Montréal, Montréal, Quebec H3T 1J4, Canada,; the § Research Centre, Saint-Justine Hospital, University of Montréal, Montréal, Quebec H3T 1C5, Canada,; the ¶ Department of Pathology and Cell Biology, University of Montréal, Montréal, Quebec H3T 1J4, Canada, and; ‖ INSERM, UMR 1048, Institut des Maladies Métaboliques et Cardiovasculaires, Université de Toulouse, F-31432 Toulouse, France

**Keywords:** arrestin, receptor, ligand, bioluminescence resonance energy transfer (BRET), chemokine, receptor endocytosis, receptor recycling, ACKR3, CXCR7, CXCL11, CXCL12, atypical chemokine receptor, chemokine scavenging, I-TAC, SDF-1, arrestin recruitment, structure-function, receptor transport

## Abstract

The atypical chemokine receptor ACKR3 contributes to chemotaxis by binding, internalizing, and degrading the chemokines CXCL11 and CXCL12 to shape and terminate chemotactic gradients during development and immune responses. Although unable to trigger G protein activation, both ligands activate G protein–independent ACKR3 responses and prompt arrestin recruitment. This offers a model to specifically study ligand-specific receptor conformations leading to G protein–independent signaling and to functional parameters such as receptor transport and chemokine degradation. We here show chemokine specificity in arrestin recruitment, by different effects of single amino acid substitutions in ACKR3 on arrestin in response to CXCL12 or CXCL11. Chemokine specificity in receptor transport was also observed, as CXCL11 induced faster receptor internalization, slower recycling, and longer intracellular sojourn of ACKR3 than CXCL12. Internalization and recycling rates of the ACKR3 R142^3.50^A substitution in response to each chemokine were similar; however, ACKR3 R142^3.50^A degraded only CXCL12 and not CXCL11. This suggests that ligand-specific intracellular receptor transport is required for chemokine degradation. Remarkably, the failure of ACKR3 R142^3.50^A to degrade CXCL11 was not caused by the lack of arrestin recruitment; rather, arrestin was entirely dispensable for scavenging of either chemokine. This suggests the involvement of another, yet unidentified, ACKR3 effector in scavenging. In summary, our study correlates ACKR3 ligand-specific conformational transitions with chemokine-dependent receptor transport dynamics and points toward unexpected ligand specificity in the mechanisms of chemokine degradation.

## Introduction

Atypical chemokine receptors (ACKRs)^6^ are devoid of G protein signaling and do not directly mediate chemotaxis. However, they modulate, shape, and extinguish chemokine gradients by virtue of efficient chemokine scavenging and degradation (1). Chemokine gradient regulation by ACKRs is crucial for the timely coordination of chemotaxis during development and the immune response. ACKRs cycle between the cell surface and intracellular compartments; they internalize chemokines, which is followed by the rapid release of chemokine degradation debris into the extracellular space (2). How and where intracellular chemokine degradation occurs remains ill-described.

ACKR3 (also known as CXCR7) binds and degrades the constitutively expressed chemokine CXCL12, which plays essential roles in embryonic organogenesis and leukocyte maturation (3–5). Chemotactic responses to CXCL12 are mediated by the canonical receptor CXCR4. ACKR3 also binds and degrades the interferon-regulated inducible chemokine CXCL11, which directs activated T-cells to target tissues during infections and in autoimmune disease, mediated by the canonical receptor CXCR3 (3, 6–8). Although lacking G protein signaling, ACKR3 recruits the scaffolding protein arrestin in response to ligand binding and activates G protein–independent signaling cascades (9, 10). Therefore, ACKR3 has been called a “biased receptor” (11).

Canonical seven-transmembrane domain receptors (7TMRs) activate both G protein and arrestin pathways, which extensively cross-talk (12), and most studies reporting agonist bias compare efficacy in G protein activation with arrestin recruitment. The extensive ligand/receptor promiscuity in the chemokine system has indeed served to illustrate agonist bias using endogenous ligands (13–15). It appears, however, that the G protein *versus* arrestin comparison is an oversimplification of the concept of agonist bias. For example, arrestin recruitment can occur following different modalities (16–19), which may entail arrestin association with different binding partners for signaling and receptor transport (20). In addition, a number of studies support the existence of signaling and receptor transport pathways that depend on neither G protein nor arrestin as effectors (21–25).

The ligand promiscuity of the chemokine system extends to ACKRs, which permits us to specifically address ligand differences among G protein–independent pathways; such differences may pertain to receptor transport and/or chemokine degradation. This also provides an opportunity to interrogate the link between ACKR arrestin recruitment, transport, and chemokine degradation, which remains a matter of debate (26–29).

In this study, we report ACKR3 substitution mutants and compare their effect on arrestin responses to the endogenous ligands CXCL11 and CXCL12. We report, as functional correlates of these different receptor conformations, receptor internalization, recycling, and chemokine degradation, using ACKR3 and the substitution mutant R142^3.50^A.

## Results

### Design and characterization of ACKR3 activation mutants

**Figure 1.**
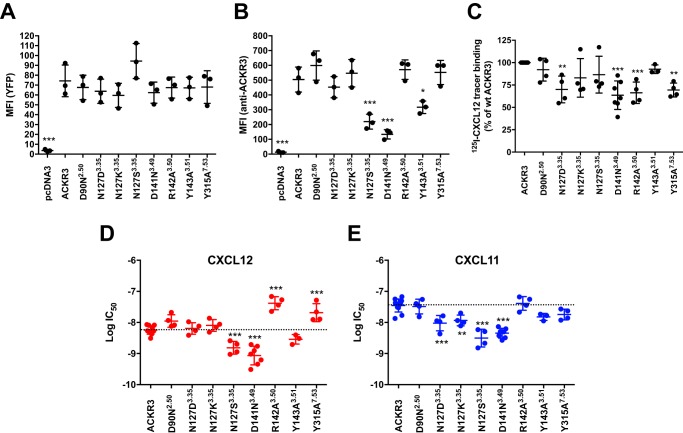
**Expression and chemokine binding of substitution mutants.** *A*, overall expression levels of mutants, measured by quantification of the C-terminal YFP tag by flow cytometry. *B*, cell surface expression levels of mutants, detected by flow cytometry after labeling with the anti-ACKR3 monoclonal antibody 358426 coupled to allophycocyanin; *C*, specific ^125^I-CXCL12 radiolabel binding to cells expressing wildtype ACKR3 or mutants. *D* and *E*, radioligand displacement using unlabeled CXCL12 (*D*, *red symbols*) or CXCL11 (*E*, *blue symbols*) on cells expressing wildtype ACKR3 or mutants. Values are means from at least three independent experiments; errors are given as S.D. (*error bars*); ANOVA with Dunnett's post hoc test: *, *p* < 0.05; **, *p* < 0.01; ***, *p* < 0.001.

Substitution mutants were designed of residues that line the activation-relevant polar network lining the central channel/cavity of the receptor and are involved in canonical 7TMR activation (G protein and arrestin recruitment) (30). The conserved positions Asp^2.50^ in TM2 (transmembrane domain 2), Asn^3.35^ in TM3, and Tyr^7.53^ in TM7 (superscript designates Ballesteros-Weinstein nomenclature (31)) were substituted, yielding mutants D90^2.50^N, N127^3.35^D, N127^3.35^K, N127^3.35^S, and Y315^7.53^A. In addition, the conserved DR^3.50^Y motif was mutated (D141^3.49^N, R142^3.50^A, and Y143^3.51^A). Overall and cell surface expression of C-terminally YFP-fused ACKR3 and mutants in transfected HEK293 cells were tested. Moreover, specific ^125^I-CXCL12 radiolabel tracer binding (50 pm) was compared between wildtype and mutant receptors, as well as the IC_50_ of competing unlabeled CXCL12 or CXCL11, as proxies for ligand affinity. As shown in Fig. 1*A*, quantification of the YFP tags suggested similar overall expression levels and thus stability of all mutants. However, mutants N127S, D141N, and Y143A were less detected at the cell surface, suggesting altered steady-state surface expression levels (Fig. 1*B*). Nevertheless, all transfectants showed substantial specific ^125^I-CXCL12 tracer binding (Fig. 1*C*) that was sufficient to perform binding competition experiments (Fig. 1 (*D* and *E*) and Table 1). Affinity of R142A and Y315A was decreased for CXCL12, but not for CXCL11, whereas N127D and N127K slightly increased CXCL11 competition (and thus affinity) but did not change CXCL12 IC_50_. Taken together, all mutants were expressed at the cell surface to some degree, competent to bind ^125^I-CXCL12 at trace concentrations, and the substitutions had different effects on the IC_50_ of the two chemokines.

**Table 1:** **Binding and activation data of ACKR3 mutants** One-way ANOVA with Dunnett's post hoc test is shown in the footnotes (boldface italic type indicates significance). ND, not determined.

Receptor/mutant	Residue number	^125^I-CXCL12 tracer binding (% of ACKR3)	CXCL12				CXCL11			
			IC_50_	Log IC _50_ ± S.D.	EC_50_	Log EC _50_ ± S.D.	IC_50_	Log IC _50_ ± S.D.	EC_50_	Log EC _50_ ± S.D.
		%	*nm*		*nm*		*nm*		*nm*	
ACKR3		100	5.9	−8.2 ± 0.1	7.0	−8.2 ± 0.2	39.1	−7.4 ± 0.2	9.8	−8.1 ± 0.2
D90N	2.50	92 ± 13	12.1	−7.9 ± 0.2	247	**−*6.7* ± *0.3**^a^***	35.9	−7.5 ± 0.2	ND	ND
N127D	3.35	***70* ± *15**^b^***	10.9	−8.2 ± 0.2	226	**−*6.6* ± *0.04**^a^***	***6.6**^a^***	**−*8.0* ± *0.2**^a^***	***906**^a^***	**−*6.1* ± *0.1**^a^***
N127K	3.35	83 ± 21	9.9	−8.1 ± 0.2	ND	ND	***11.0**^a^***	**−*7.9* ± *0.2**^b^***	ND	ND
N127S	3.35	87 ± 21	1.7	**−*8.8* ± *0.2**^a^***	ND	ND	***3.7**^a^***	**−*8.5* ± *0.3**^a^***	ND	ND
D141N	3.49	***64* ± *16**^a^***	1.0	**−*9.1* ± *0.3**^a^***	ND	ND	***4.8**^a^***	**−*8.3* ± *0.2**^a^***	ND	ND
R142A	3.50	***66* ± *12**^a^***	***45.1**^a^***	**−*7.4* ± *0.2**^a^***	***585**^a^***	**−*6.2* ± *0.1**^a^***	44.8	−7.4 ± 0.2	ND	ND
Y143A	3.51	93 ± 5	3.0	−8.5 ± 0.1	***493**^b^***	**−*6.4* ± *0.3**^a^***	***15.4**^c^***	−7.8 ± 0.1	***436**^c^***	**−*6.4* ± *0.3**^a^***
Y315A	7.53	***69* ± *8**^b^***	***24.7**^b^***	**−*7.7* ± *0.3**^a^***	ND	ND	***19.1**^c^***	−7.7 ± 0.2	ND	ND

*^a^ p* < 0.001.

*^b^ p* < 0.01.

*^c^ p* < 0.05.

**Figure 2.**
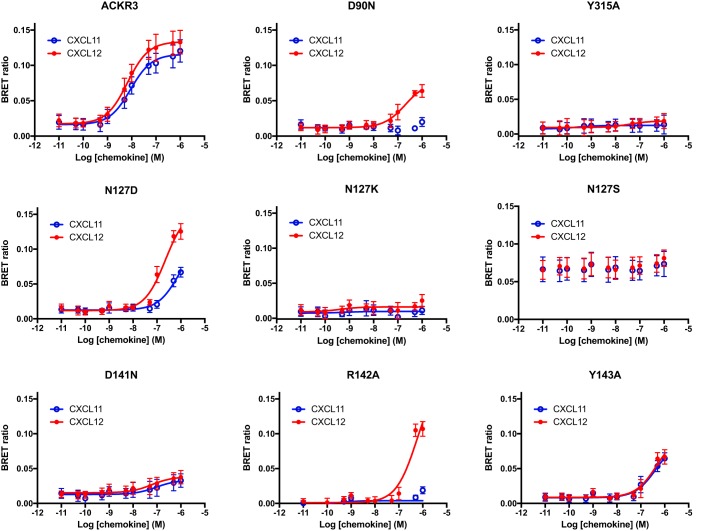
**Arrestin recruitment to ACKR3 mutants.** Shown are dose-response curves of arrestin recruitment to wildtype ACKR3 (*top left*) or the indicated mutants after CXCL12 (*red lines* and *symbols*) or CXCL11 (*blue lines* and *symbols*) challenge. The maximal ligand concentration used was 1 μm. Values are means from at least three independent experiments conducted in duplicate. *Error bars*, S.D.

Arrestin responses to CXCL11 and CXCL12 were tested using bioluminescence resonance energy transfer (BRET), in dose-response experiments using up to 1 μm ligand, as before (9, 29). All substitutions affected arrestin recruitment at least quantitatively (Fig. 2 and Table 1). Most strikingly, mutants D90N and R142A were still competent to recruit arrestin in response to CXCL12 (albeit reduced) but completely abolished CXCL11 responses. This highlights differences in the conformational transitions that trigger arrestin recruitment in response to the two ligands and that most likely derive from their different binding modes to the receptor (29). Substitutions N127K, N127S, D141N, and Y315A abolished all ligand responses. Mutant N127S yielded 5-fold increased basal BRET, suggesting constitutive arrestin recruitment. Constitutive activation of this mutant may thus also underlie the observed reduced surface expression of this mutant and its increased agonist affinity (Fig. 1 and Table 1). Of note, in CXCR4, substitution of Asn^3.35^ by serine leads to constitutive G protein signaling (32) (whereas arrestin recruitment is not yet reported for this CXCR4 mutant).

### Chemokine scavenging by ACKR3 mutants

**Figure 3.**
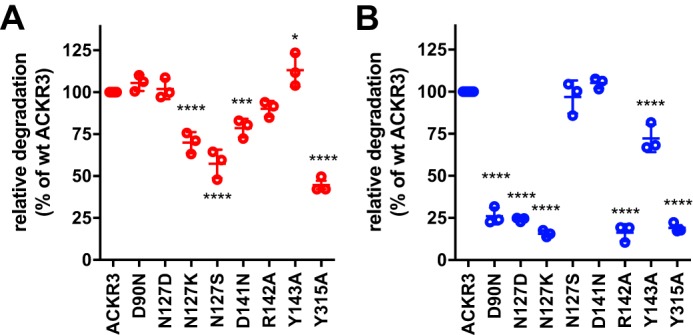
**Chemokine scavenging by ACKR3 mutants.** Relative degradation of ^125^I-CXCL12 (*A*, *red symbols*) or of ^125^I-CXCL11 (*B*, *blue symbols*) by cells expressing wildtype or mutant ACKR3 fused to YFP. Cells were transfected with 10 ng (*A*) or 100 ng (*B*) of coding DNA/well (6-well plates). Receptor expression was monitored by flow cytometry via the YFP tag; expression levels of impaired mutants were not below expression levels of wildtype ACKR3, and expression levels with intact scavenging did not exceed expression levels of wildtype ACKR3. Values are from at least three independent experiments conducted in duplicate. *Error bars*, S.D.; ANOVA with Dunnett's post hoc test: *, *p* < 0.05; **, *p* < 0.01; ***, *p* < 0.001; ****, *p* < 0.0001.

The impact of the substitutions on chemokine ligand scavenging, one of the main ACKR3 functions, was tested. Degradation of ^125^I-chemokines at trace concentrations (50 pm) was measured using cells transfected with minute amounts of receptor plasmid, to avoid saturation of scavenging by receptor overexpression, as previously established (29); mutant expression levels were controlled by quantification of the signal of the C-terminal YFP tag. As shown in Fig. 3, the substitutions that affected scavenging clearly differed between CXCL12 and CXCL11. Substitutions N127S and D141N only reduced CXCL12, but not CXCL11 scavenging, whereas D90N, N127D, and Y143A reduced CXCL11 but not CXCL12 degradation. Scavenging of both chemokines was affected by the N127K and Y315A substitutions. Therefore, different determinants govern degradation of each chemokine. Strikingly, chemokine degradation efficiency and arrestin responses did not correlate, despite some overlap (*e.g.* compare CXCL11 arrestin response and degradation of mutants N127D and D141N), in line with our previous observations (29). This prompted us to further investigate the role of arrestin for ACKR3-mediated ligand scavenging.

### Chemokine scavenging by ACKR3 does not require arrestin

**Figure 4.**
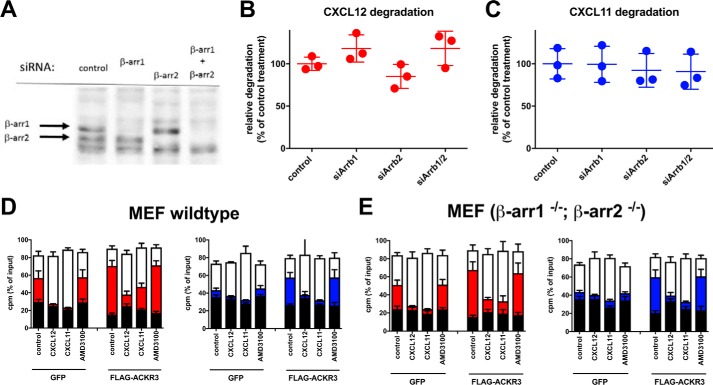
**Arrestin is not required for chemokine degradation by ACKR3.** *A*, Western blotting of lysates transfected with siRNA targeting β-arrestin1, β-arrestin2, or both arrestin isoforms, using pan-arrestin antibody. *B* and *C*, degradation of ^125^I-CXCL12 (*B*) or ^125^I-CXCL11 (*C*) is unaffected by arrestin knockdown with siRNA. *D* and *E*, MEFs from wildtype (*D*) or β-arr1^−/−^/β-arr2^−/−^ double knock-out (*E*) animals were transduced with lentivirus coding for GFP (control) or hACKR3, as indicated. Degradation of ^125^I-CXCL12 (*red symbols*) or ^125^I-CXCL11 (*blue symbols*) was determined; *stacks* show undegraded chemokine (*top*, *white fields*), degraded chemokine debris (*middle*, *colored fields*), and cell-associated chemokine (*bottom*, *black fields*). Degradation was blocked using 200 nm of unlabeled chemokine or 1 μm AMD3100, as indicated. *A*, one representative experiment of three independent experiments; *B–E*, pooled data of three independent experiments conducted in duplicate. *Error bars*, S.D.

The effect of siRNA knockdown of arrestin in HEK293 cells on chemokine degradation was thus tested. As shown in Fig. 4, complete knockdown of arrestin on the protein level, as judged by Western blotting, did not significantly affect scavenging of either chemokine (Fig. 4, *A–C*). However, siRNA knockdown does not entirely rule out ongoing expression of minute amounts of arrestin that might suffice for scavenging. We thus also tested genetically arrestin-ablated murine embryonic fibroblasts (MEFs), infected with lentivirus encoding green fluorescent protein (control) or human ACKR3 (hACKR3), as a second line of evidence. Wildtype MEFs infected with control virus readily scavenged CXCL12; CXCL12 scavenging was further increased upon overexpression of hACKR3 (Fig. 4*D*). In contrast, control-infected wildtype MEFs scavenged CXCL11 only weakly, but this was drastically increased by overexpression of hACKR3. In all cases, scavenging was reduced by competition with unlabeled CXCL11 or CXCL12, indicating that MEFs express sufficient endogenous murine ACKR3 for CXCL12, but not for CXCL11, degradation. Inefficient scavenging of human CXCL11 by endogenous murine ACKR3 may reflect low affinity of the murine receptor for the human chemokine (human and murine CXCL12 are almost identical, unlike human and murine CXCL11). Perhaps unexpectedly, very similar results were obtained with double-knockout MEFs devoid of both β-arrestin isoforms (Fig. 4*E*). Taken together, these data demonstrate that arrestin is indeed dispensable for chemokine scavenging by ACKR3. In consequence, the failure of ACKR3 mutants to recruit arrestin cannot explain their failure to degrade CXCL12 or CXCL11, in line with the lack of correlation between the two parameters (Fig. 3 and Ref. 29). Rather, failure to bind and/or activate other, unidentified chemokine-specific downstream interactors and/or effectors must therefore account for the observed inability to degrade chemokines. It is probable that such ligand-specific interactors/effectors relate to receptor internalization and intracellular transport.

### Chemokine-dependent differences in ACKR3 intracellular trafficking

**Figure 5.**
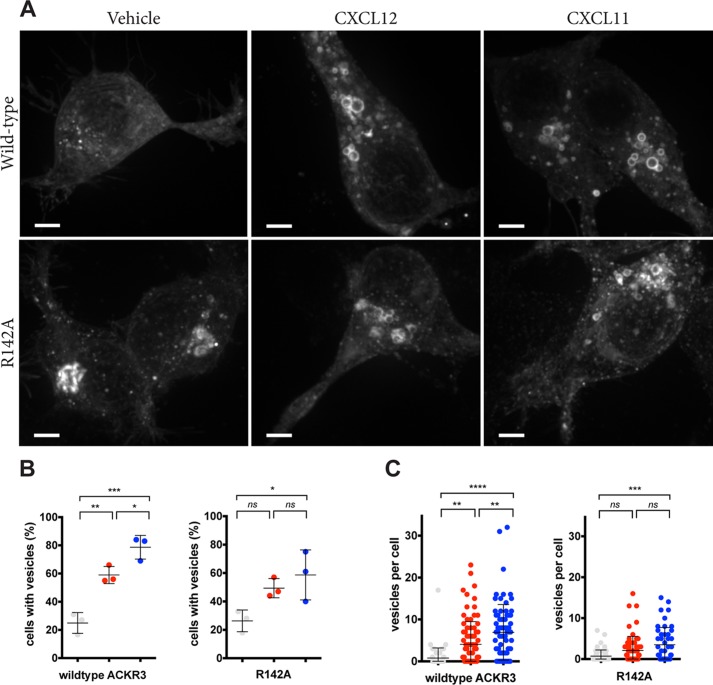
**ACKR3-YFP accumulation in vesicular compartments after chemokine stimulation.** *A*, HEK239 cells expressing wildtype or R142A ACKR3 fused to YFP were followed by spinning disc microscopy during stimulation with 200 nm CXCL12 or CXCL11. Representative confocal images (maximum intensity projections) after 1 h of stimulation are shown. *Scale bars*, 4 μm. *B*, the percentage of cells accumulating YFP in vesicles; *C*, the number of fluorescent vesicles per cell. *Gray symbols*, vehicle; *red symbols*, CXCL12; *blue symbols*, CXCL11. Data are from three independent experiments; *error bars*, S.D.; ANOVA with Turkey's post hoc test: *, *p* < 0.05; **, *p* < 0.01; ***, *p* < 0.001; ****, *p* < 0.0001.

To further address potential ligand-specific differences in ACKR3 trafficking, receptor redistribution in response to the two chemokines was studied in live cells. Wildtype ACKR3 was compared with mutant R142A, which showed a ligand-dependent effect on arrestin recruitment and scavenging. HEK293 cells were transfected with receptor-YFP fusion proteins, and receptor redistribution after chemokine stimulation was observed by spinning disc confocal microscopy. Without chemokine stimulation, most of the receptor was localized inside the cells in diffuse freckles, as reported previously (33) (Fig. 5*A*). Chemokine challenge led to receptor redistribution into round vesicles in 60–80% of the cells; this was observed in only 25% of the unstimulated cells (Fig. 5*B*). When stimulated with CXCL11, significantly more cells accumulated vesicles compared with CXCL12 (*p* < 0.05). Moreover, the number of receptor-YFP decorated vesicles per cell increased after chemokine challenge (Fig. 5*C*); again, more vesicles accumulated per cell with CXCL11 stimulation compared with CXCL12 (*p* < 0.01). The R142A substitution attenuated both parameters, and the differences between CXCL11 and CXCL12 became insignificant. These data suggest that more wildtype ACKR3, but not R142A, accumulated in vesicles after CXCL11 challenge, compared with CXCL12. It is of note that CXCL11-increased intracellular ACKR3 accumulation cannot be caused by higher affinity, because the CXCL11 affinity to ACKR3 is inferior to that of CXCL12. Rather, our observations suggest qualitatively different responses.

**Figure 6.**
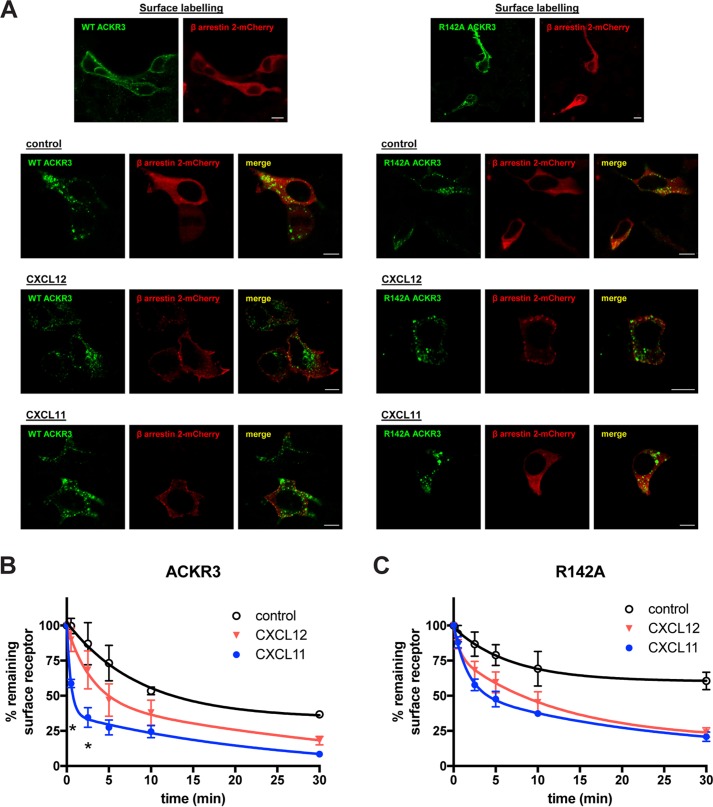
**Internalization of prelabeled surface ACKR3.** *A*, confocal microscopy of HEK293 cells co-expressing wildtype or mutant ACKR3 (bearing an N-terminal FLAG tag but no C-terminally fused YFP tag) and β-arrestin2 mCherry (*red fluorescence*). The *top row* shows surface labeling of the N-terminal FLAG tag and revelation with secondary Alexa Fluor® 488-conjugated antibody (*green fluorescence*) at 4 °C. The *other rows* show secondary labeling of permeabilized cells after 90 min of rapid temperature shift to 37 °C in the absence (control) or presence of 100 nm CXCL12 or CXCL11. *B* and *C*, quantification by flow cytometry of receptor internalization after prelabeling with uncoupled anti-FLAG antibody, followed by temperature shift and stimulation as indicated. Secondary labeling was performed at the indicated time points. Upon simultaneous curve fit, the preferred fit is a two-phase decay for ACKR3/CXCL11 (*p* < 0.0001), but also R142A/CXCL12 (*p* = 0.029) and R142A/CXCL11 (*p* = 0.0010), whereas a one-phase decay model is preferred in the absence of stimulation and for ACKR3/CXCL12 (ANOVA with Tukey's post hoc test at the indicated time point: *, *p* < 0.05 between CXCL11 and CXCL12). All data are from three independent experiments, and *error bars* represent S.D.

To specifically follow surface-exposed receptor, and to concomitantly follow arrestin relocalization, we performed confocal microscopy. N-terminally FLAG-tagged ACKR3 was co-expressed with β-arrestin2 fused to the red fluorescent protein mCherry (34). Surface receptor was decorated with unlabeled anti-FLAG antibody at 4 °C before washing the cells and re-exposure to 37 °C, with and without stimulation, followed by staining with fluorescence-labeled secondary antibody. As shown in Fig. 6 (*A* and *B*), ACKR3 internalized regardless of the presence of chemokine, as described previously (33). Relocalization of co-expressed β-arrestin2-mCherry, however, depended on the presence of chemokines. The R142A mutant did not relocalize arrestin in response to CXCL11 (but did so in response to CXCL12), confirming the inability of the mutant to recruit arrestin in response to CXCL11, which was previously observed by BRET (Fig. 2). Similar results were obtained after 30 and 60 min of stimulation (Fig. S1). Of note, relocalized arrestin did not always co-localize with internalized receptor, but was also observed in patches at the plasma membrane (see also Fig. S1), regardless of the chemokine used, reminiscent of similar observations made in the β-adrenergic receptor system (35). The R142A/CXCL11 images also confirm that receptor internalization, like chemokine scavenging, can occur independently from arrestin recruitment.

To quantify receptor internalization kinetics, we performed flow cytometry and measured the proportion of prelabeled surface receptor that remained at the surface at different time points. As shown in Fig. 6*B*, CXCL11 induced significantly faster internalization of wildtype ACKR3 than CXCL12. Intriguingly, internalization kinetics after CXCL11, but not CXCL12, challenge fitted biphasic decay best (*p* < 0.0001), involving a very rapid initial phase of internalization during the first few minutes after stimulation. Accordingly, remaining surface receptor differed significantly at very early time points between the chemokines (*p* < 0.05, *t* test). Again, given the higher affinity of ACKR3 for CXCL12 relative to CXCL11, it is unlikely that affinity differences account for the faster internalization after CXCL11 exposure. Interestingly, the fast component of receptor internalization after CXCL11 challenge was strongly attenuated by the R142^3.50^A substitution, and internalization rates of this mutant were similar after challenge with either chemokine.

Receptor recycling was then addressed, using a protocol first used for ACKR3 by Naumann *et al.* (33). After chemokine stimulation for 15 min at 37 °C, the remaining chemokine and surface receptor were degraded with proteinase K on ice. This was followed by re-exposure of the cells to 37 °C, and time-dependent reappearance of receptor at the cell surface was quantified by staining with fluorescence-labeled anti-ACKR3 antibody. Of note, preincubation and the continuous presence of cycloheximide during these experiments ensured that reappearing surface receptor was indeed recycled and not newly synthesized.

**Figure 7.**
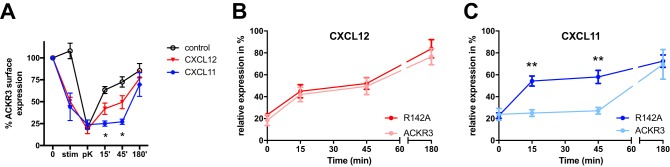
**Differences in ACKR3 recycling after stimulation with CXCL12 or CXCL11 are abolished in the R142A mutant.** *A*, direct labeling of surface receptor with anti-ACKR3 antibody 358426 coupled to APC. Transfected HEK293 cells were preincubated for 2 h with 50 μm cycloheximide (*0*). They were then stimulated for 15 min with vehicle (control) or 200 nm chemokines (*stim*), before digestion of remaining surface receptor and chemokine with proteinase K on ice (*pK*). The cells were then washed and shifted to 37 °C for 15, 45, or 180 min. Note the absence of rapid reappearance of ACKR3 after CXCL11 stimulation at 15 and 45 min, but not 180 min (ANOVA with Tukey's post hoc test at the indicated time point: *, *p* < 0.05 between CXCL11 and CXCL12). *B* and *C*, recycling rates of R142A (*bold color*) compared with wildtype ACKR3 (*pale color*), after stimulation with CXCL12 (*B*, *red symbols*) or CXCL11 (*C*, *blue symbols*). The time point *0* in *B* and *C* corresponds to the point *pK* in *A*. **, *p* < 0.01, *t* test between ACKR3 and R142A. All data are from three independent experiments. *Error bars*, S.D.

As shown in Fig. 7, no recycling was observed at earlier time points after CXCL11 challenge, in contrast to significant recycling after 15 and 45 min following stimulation with CXCL12. Nevertheless, the proportion of recycled receptor was similar after 3 h, in line with a previous report (36). The significant delay in ACKR3 recycling after CXCL11 challenge was abrogated by the R142^3.50^A substitution mutant, for which receptor recycling rates did not significantly differ after CXCL12 or CXCL11 stimulation (Fig. 7, *B* and *C*).

Taken together, these data demonstrate that ACKR3 internalization and recycling rates are different after exposure to CXCL12 or CXCL11. CXCL11 leads to more rapid internalization (Fig. 6*B*) and delayed recycling (Fig. 7). Both mechanisms should prolong intracellular sojourn of the receptor, providing independent support for the conclusions drawn from the observations made in Fig. 5. Strikingly, in line with the microscopy results, the R142A substitution abrogated these particularities. Indeed, after CXCL11 challenge of R142A, the quantity of vesicle-associated receptor and the receptor internalization and recycling rates are close to those observed with CXCL12-challenged wildtype ACKR3. Nevertheless, despite matching kinetics, R142A degrades only CXCL12 and not CXCL11 (Fig. 3). Taken together, these independent lines of evidence suggests that the characteristic ACKR3 transport kinetics in response to CXCL11 are mandatory for its degradation. Indeed, receptor internalization and recycling kinetics that permit CXCL12 degradation do not lead to CXCL11 degradation, pointing toward chemokine-specific intracellular transport as a prerequisite for efficient scavenging.

## Discussion

Progress in the structural biology of 7TMRs has begun to unravel the fine-tuning of the conformational changes by which receptors translate extracellular ligand binding into intracellular interactions with different effectors (37–39). ACKR3, which has been called a “biased receptor” (11), permits comparison of the effects of point mutations on conformational transitions that specifically lead to activation of G protein–independent signaling in response to two different ligands. Our substitutions targeted conserved polar residues that line the central water-mediated polar network, which undergoes major rearrangements upon activation (40), also in ACKR3 (41). Importantly, in canonical 7TMRs, substitution of different positions lining the network affects G protein and arrestin signaling differently, implying that this region relates to the fine-tuning of coupling efficiencies to different effectors.

We find that, in ACKR3, mutation N127^3.35^K blocks arrestin recruitment induced by either CXCL12 or CXCL11, in line with the N^3.35^K substitution keeping TM3 in a fixed position, thus globally preventing ligand-induced conformational changes in the receptor (37). Inversely, constitutive arrestin recruitment to the N127^3.35^S mutant suggests spontaneous release from conformational constraints of the inactive receptor state. Indeed, position Asn^3.35^ is a known central activation switch of canonical chemokine, angiotensin, and opioid receptors, where substitutions with small residues result in constitutive activity, whereas substitution with lysine freezes the receptor in the inactive state (32, 42, 43). Our findings thus suggest a similar role for position Asn^3.35^ in ACKR3 and indicate no fundamental difference from the overall activation mechanisms of canonical 7TMRs.

Our data highlight, however, that ACKR3 ligation by CXCL11 or CXCL12 results in different conformational transitions. Mutation of the highly conserved D90^2.50^N and R142^3.50^A were deleterious for the arrestin response to CXCL11 but still permitted substantial (although reduced) responses to CXCL12. In canonical 7TMRs, substitutions of the conserved DR^3.50^Y motif affect G protein and arrestin pathways differently (44), and Arg^3.50^ may indeed directly participate in interaction not only with the G protein C terminus (45, 46), but also with the arrestin finger loop in the “core” configuration of arrestin recruitment (47, 48). Our data are compatible with qualitatively different engagement of the central receptor cavity by arrestin after stimulation with the different chemokines. Mutation of residue Asp^2.50^ results in reduced G protein activation by various 7TMRs (40, 42, 49), potentially also through increased efficacy of arrestin coupling (50, 51); however, blunting of arrestin responses by the Asp^2.50^ substitution was also reported (52). This suggests that the Asp^2.50^ role for arrestin recruitment depends on the receptor and the ligand being studied. Finally, substitution of position Tyr-315^7.53^, which is not involved in G protein activation, completely blocked arrestin recruitment to ACKR3 by both chemokines. This is consistent with its reported central role for arrestin responses of canonical 7TMRs (49, 50, 53–55).

Taken together, several overall features (*i.e.* the roles of Asn^3.35^ and Tyr^7.53^) of the ACKR3-activating conformational transitions resemble those of the canonical 7TMRs. Our data also provide evidence for different ACKR3 conformational transitions following CXCL11 or CXCL12 binding, consistent with the different receptor-binding modes of these chemokines (29). This suggests that different sets of effectors may be engaged, and the same effector may be engaged following different modes of interaction, in response to the two ligands. This may entail different signaling and/or receptor transport.

Indeed, our data consistently show receptor transport differences in that CXCL11, compared with CXCL12, induces faster ACKR3 internalization and slower recycling, leading to longer intracellular sojourn and accumulation of the receptor. This is not explained by differences in ligand affinity or potency, because CXCL11 has lower affinity than CXCL12, but points toward different mechanisms of endocytosis and intracellular transport. Ligand-dependent endocytosis and recycling rates of the same receptor have been reported for canonical chemokine receptors (14, 56). Our data suggest that these previous observations may not be simply due to different arrestin recruitment efficacies or to differentially interfering or cross-talking G protein signaling at canonical receptors but also to qualitative differences among the engaged G protein–independent pathways.

With the ACKR3 R142A substitution mutant, internalization and recycling rates in response to CXCL11 and CXCL12 were matched and resembled those of the wildtype receptor after CXCL12 challenge. Nevertheless, ACKR3 R142A degraded CXCL12, but not CXCL11 (for which its affinity is unaltered), indicating that the characteristic receptor transport kinetics after CXCL11 challenge are necessary for degradation of this chemokine. In other words, chemokine-specific receptor transport was a requirement for degradation and may relate to individual intracellular degradation compartments and/or mechanisms for each chemokine. Consistent with different requirements for degradation, CXCL11 degradation was severely blunted by substitutions D90N, N127D, and R142A, which did not affect CXCL12 degradation. Inversely, substitutions N127S and D141N affected CXCL12 but not CXCL11 degradation. Of note, the sets of mutants affecting chemokine degradation did not match arrestin recruitment phenotypes, despite some overlap (such as for R142A), as already observed previously (29). Our data do not exclude a subtle role for arrestin in chemokine degradation by modulation of the intracellular receptor transport, as reported with the formyl peptide receptor FPR (57), but also with ACKR3 (58) and ACKR2 (27). However, our results using anti-arrestin siRNA and arrestin double-knock-out MEFs exclude arrestin itself as an essential player in ACKR3 transport to specific intracellular degradation compartments and also in ACKR3 internalization, contrary to previously claims (36). Rather, the involvement of other, unidentified, ACKR3 effectors needs to be considered. The potential role of arrestin in ACKR-mediated chemokine degradation is the subject of debate, with some, but not all, investigators finding arrestin dispensable (26–28, 59). Our previous work had already highlighted that degradation efficiency and arrestin recruitment can be discordant for ACKR3 substitution mutants (29), and we now confirm that arrestin is not needed for ACKR3-mediated chemokine degradation.

Therefore, overall, our data show that ligand-dependent ways to engage arrestin, the sole known ACKR3-interacting protein, overlap with, but are not causal of, ligand-specific receptor trafficking, which is needed for chemokine degradation. This opens up a series of new questions that relate to (i) ligand-specific ACKR3 engagement of other, unknown adaptor proteins, (ii) the diverging intracellular transport between CXCL11- and CXCL12-bound ACKR3, and (iii) the requirements for efficient CXCL11 degradation. Indeed, additional effectors and/or adaptor proteins that ACKR3 can engage in a ligand-specific way need to be identified. Arrestin domain-containing proteins, which reportedly modulate receptor trafficking and endosomal residence time (60), may be promising candidates. However, a host of other candidates exist, such as members of the NHERF family (61). It also needs to be clarified whether ligand-specific ACKR3 adaptors direct CXCL11- and CXCL12-bound ACKR3 toward separate endocytic and transport pathways, and if so, the resulting respective intracellular trajectories need to be delineated in more detail. Alternatively, ligand-specific adaptor proteins might instead regulate the dynamics of the same endocytic machinery, as described previously (62). Finally, it remains an open question whether the requirement for CXCL11-specific intracellular receptor transport for efficient degradation of this chemokine reflects the intervention of specific sets of enzymes that reside in defined intracellular compartments. In this case, access to such CXCL11-degrading compartments would depend on adaptors that are specifically engaged by CXCL11-bound ACKR3. Alternatively, the intracellular degradation kinetics of CXCL11 may be significantly slower than those of CXCL12 or require different pH but occur essentially via the same mechanisms; this might also explain the need for longer intracellular sojourn of CXCL11-bound ACKR3 for efficient degradation. More research is needed to distinguish between these possibilities.

## Experimental procedures

### Materials

Recombinant chemokines were a kind gift from Amanda Nevins and Brian Volkman or from PeproTech Inc. (Rocky Hill, NJ); radiolabeled chemokines were from PerkinElmer Life Sciences. Coelenterazine h (for BRET1 experiments) was from NanoLight Technology (Pinetop, AZ). The anti-ACKR3 (CXCR7) monoclonal antibodies (clones 358462 and 11G8) as well as allophycocyanin (APC)-labeled goat anti-mouse antibody were from R&D Systems (Minneapolis, MN), and anti-FLAG M2 antibody was from Sigma.

### Generation of ACKR3 mutants

Receptor mutants were generated by PCR in ACKR3-YFP, which has been described and analyzed before (9), and in ACKR3 bearing an N-terminal FLAG tag; the sequences of the resulting mutants were confirmed by sequencing.

### Cell culture and transfection

HEK293 cells were maintained in Dulbecco's modified Eagle's medium (DMEM), 1% penicillin-streptomycin (Invitrogen), and 10% fetal bovine serum (Wisent, Montreal, Quebec, Canada). Transient transfections were performed using the polyethyleneimine method.

### Flow cytometry

Receptor mutant expression was quantified by flow cytometry. Cells were stained for 30 min on ice, followed by washing and analysis using a FACSCalibur (BD Biosciences) cytometer. Overall mutant protein expression was quantified by the C-terminal YFP moiety. Data analysis was performed using CellQuestPro and FlowJo software.

### Radioligand-binding assays

HEK293 cells expressing ACKR3-YFP or the indicated mutants were incubated for 5 min at 37 °C with 100 μm phenylarsine oxide. Cells were collected, washed, and incubated with 50 pm ^125^I-CXCL12 tracer in binding buffer (50 mm HEPES, pH 7.4, 5 mm MgCl_2_, 1 mm CaCl_2_, 0.2% BSA) in the presence or absence of the indicated concentrations of unlabeled CXCL12 or CXCL11 for 30 min at 37 °C. After removal of unbound tracer, bound radiolabel was quantified in a γ counter.

### Arrestin recruitment

β-Arrestin2 recruitment was monitored as described previously using a BRET-based assay (10). Briefly, HEK293 cells were transiently transfected with receptor-YFP fusion and β-arrestin2-Rluc constructs. Transfected cells were seeded onto poly-d-lysine–treated 96-well plates. Culture medium was replaced with PBS supplemented with 0.1% BSA and 0.5 mm MgCl_2_ at 48 h post-transfection. Cells expressing -YFP and -Rluc fusions at a ratio resulting in BRET_max_ were stimulated with chemokine ligands for 5 min at 37 °C followed by the addition of coelenterazine h to 5 μm (NanoLight Technology) for 10 min at 37 °C. Fluorescence and luminescence readings were performed using a Mithras LB940 plate reader (Berthold Technologies, Bad Wildbad, Germany) while the cells remained attached to the plastic surface of the assay plates. BRET_net_ is calculated by subtracting the background BRET ratio observed in cells expressing β-arrestin2-Rluc alone.

### Chemokine scavenging

Chemokine degradation was essentially performed as before (29, 63). Cells were transfected in 6-well plates with 10 or 100 ng/well plasmid (for CXCL12 or CXCL11 degradation, respectively). 24 h post-transfection, cells were transferred to 24-well plates and allowed to attach. Receptor mutant expression levels were quantified by flow cytometry, monitoring mean fluorescence intensity in the YFP channel; only mutant expression levels greater than or equal to that of wildtype ACKR3 were used for degradation-impaired mutants, and less than or equal to that of wildtype ACKR3 for degradation-intact mutants. For degradation, medium was replaced with 200 μl of 50 pm ^125^I-labeled chemokine in HEPES-buffered serum-free DMEM containing 1% BSA for 3 h at 37 °C. Supernatants were collected, and cells were washed in glycine buffer (pH 2.8) to remove surface-attached chemokine; washing buffer was pooled with supernatant and subjected to 12.5% TCA precipitation. Precipitate represents intact chemokine, whereas the supernatant contains chemokine degradation products; radioactivity that remained associated with the cells was also counted.

### siRNA knockdown and Western blotting

HEK293 cells were seeded into 60-mm cell culture dishes in DMEM supplemented with 10% fetal bovine serum (Gibco) and 1% penicillin-streptomycin (Invitrogen). The next day, cell culture medium was replaced by DMEM without penicillin-streptomycin. The cells were then transfected using Lipofectamine RNAiMax (Invitrogen) with 5 μl of either ARRB1 or ARRB2 siRNA (20 μm) or with both siRNAs (ON-TARGET plus ARRB1/2 siRNA- SMART pool, GE Healthcare). The transfection was repeated 24 h later without changing cell culture media. The transfected cells were maintained for another 24 h in culture in DMEM, 10% fetal bovine serum, and 1% penicillin-streptomycin. The cells were finally washed with PBS, collected, resuspended in PBS, and placed at −20 °C. Cells were frozen and lysed after 24 h or processed for the chemokine scavenging assay. Total protein concentration of cell lysates was determined using the DC protein assay (Bio-Rad). 50 μg of total protein from each cell lysate were separated by 8% polyacrylamide SDS-PAGE and transferred onto a PVDF membrane. The membrane was blocked in TBST buffer (100 mm Tris, 150 mm NaCl, 0.1% Tween, pH 8) containing 5% nonfat milk for 1 h at room temperature, followed by incubation overnight at 4 °C with a 1:500 dilution of anti-β-arrestin antibody (BD Biosciences) in TBST buffer containing 5% bovine serum albumin. After washings in TBST, membrane was incubated for 1 h at room temperature with a 1:1000 dilution of anti-mouse HRP antibody (Jackson Immunoresearch Laboratories) and again washed with TBST. Revelation was done using ECL substrate (Clarity Western ECL substrate, Bio-Rad) and digital imaging (G:BOX Chemi XRQ with Genesys software, Syngene).

### hACKR3 overexpression in MEFs

Murine embryonic fibroblasts devoid of both β-arrestin1 and β-arrestin2 expression were generous gifts from Dr. Robert Lefkowitz (Duke University Medical Center). Lentiviral vectors were based on pCCLsin-hPGK (Addgene) and expressed either GFP (control) or FLAG-tagged hACKR3. Infected cells were processed for chemokine degradation assays as described above.

### Live cell imaging

HEK293 cells transfected with wildtype or R142A ACKR3-YFP were seeded into 8-well chambered coverglass slides (Labtek). Live cell imaging was performed using a spinning-disc confocal system (UltraVIEW Vox, PerkinElmer Life Sciences) consisting of a scanning unit (CSU-X1; Yokogawa Corp.) and a charge-coupled device camera (ORCA-R2; Hamamatsu Photonics) fitted to an inverted microscope (DMI6000 B; Leica) equipped with a motorized piezoelectric heated stage (Applied Scientific Instrumentation). Image acquisition and vesicle quantification was performed using Volocity software, version 6.3 (PerkinElmer Life Sciences). Imaging was performed using a Plan Apochromat ×40, 0.85 numerical aperture air objective with camera binning set to 2 × 2. High-resolution imaging was performed using Plan Apochromat ×100 oil immersion objectives, numerical aperture 1.4, with camera binning set to 2 × 2. For each of the three independent experiments, at least five fields per condition were randomly chosen and analyzed. For each field, 10 *z*-slices of 1 μm were acquired.

### ACKR3 internalization and recycling

For ACKR3 internalization and recycling assays, FLAG-ACKR3–expressing HEK293 cells were used. To specifically follow internalization, cells were prelabeled with anti-FLAG M2 antibody (Sigma) on ice. Cells were then washed, stimulated with a 200 nm concentration of the indicated chemokine, and shifted to 37 °C. Aliquots were drawn at the indicated time points, washed, and labeled with secondary APC-coupled goat anti-mouse antibody (R&D Systems). The protocol for ACKR3 recycling was essentially as described (33), using cells cultured in DMEM supplemented with dialyzed fetal calf serum (Gibco). FLAG-ACKR3–expressing cells were exposed for 2 h to 50 μm cycloheximide in HEPES-buffered DMEM without serum but with 1% BSA before inducing receptor internalization for 15 min as above. Cells were then spun down and incubated on ice with 0.5 μg/ml proteinase K (Sigma) for 90 min to digest remaining surface receptor and chemokine. After washing, the cells were shifted to 37 °C for various intervals. At all steps of the experiments, aliquots were drawn, and surface receptor was labeled with APC-coupled anti-ACKR3 monoclonal antibody 358426. Surface ACKR3 was quantified by flow cytometry as above.

### Confocal microscopy

HEK239T cells expressing wildtype or R142A mutant FLAG-ACKR3 and β-arrestin2-mCherry were prelabeled with anti-FLAG M2 antibody (Sigma) on ice for 1 h, washed, and then stimulated with or without a 100 nm concentration of the indicated chemokine at 37 °C for 1 h. After washing in cold PBS, cells were fixed in 4% paraformaldehyde, permeabilized with 0.05% saponin, saturated with PBS containing 10% fetal bovine serum, and incubated with Alexa Fluor® 488-conjugated fluorescent secondary antibody (Life Technologies, Inc.). Coverslips were then mounted onto glass slides using Mowiol. Confocal images were captured with a LSM 780 operated with Zen software using a ×63, 1.4 numeric aperture Plan-Apochromat objective (Carl Zeiss).

### Data analysis

Data analysis was performed using GraphPad Prism version 6 software (GraphPad Software, Inc., La Jolla, CA).

## Author contributions

N. M., G. H., V. P., and N. H. designed the study; N. M., G. St-O., N. N., D. R., B. B., M. G., and V. P. performed experiments; N. M., G. St-O., N. N., D. R., B. B., M. G., V. P., and N. H. analyzed the data. N. H. wrote the manuscript with input from the other authors; all authors approved the final version of the manuscript.

## Supplementary Material

Supporting Information

## Footnotes

6 The abbreviations used are:

ACKR: atypical chemokine receptor
hACKR: human ACKR
7TMR: seven-transmembrane domain receptor
BRET: bioluminescence resonance energy transfer
MEF: mouse embryo fibroblast
TM: transmembrane domain
ANOVA: analysis of variance
DMEM: Dulbecco's modified Eagle's medium
APC: allophycocyanin.

## References

[1] Bachelerie F., Graham G. J., Locati M., Mantovani A., Murphy P. M., Nibbs R., Rot A., Sozzani S., and Thelen M. (2015) An atypical addition to the chemokine receptor nomenclature: IUPHAR Review 15. Br. J. Pharmacol. 172, 3945–3949 10.1111/bph.13182 25958743 PMC4543604

[2] Weber M., Blair E., Simpson C. V., O'Hara M., Blackburn P. E., Rot A., Graham G. J., and Nibbs R. J. (2004) The chemokine receptor D6 constitutively traffics to and from the cell surface to internalize and degrade chemokines. Mol. Biol. Cell 15, 2492–2508 10.1091/mbc.E03-09-0634 15004236 PMC404040

[3] Burns J. M., Summers B. C., Wang Y., Melikian A., Berahovich R., Miao Z., Penfold M. E., Sunshine M. J., Littman D. R., Kuo C. J., Wei K., McMaster B. E., Wright K., Howard M. C., and Schall T. J. (2006) A novel chemokine receptor for SDF-1 and I-TAC involved in cell survival, cell adhesion, and tumor development. J. Exp. Med. 203, 2201–2213 10.1084/jem.20052144 16940167 PMC2118398

[4] Boldajipour B., Mahabaleshwar H., Kardash E., Reichman-Fried M., Blaser H., Minina S., Wilson D., Xu Q., and Raz E. (2008) Control of chemokine-guided cell migration by ligand sequestration. Cell 132, 463–473 10.1016/j.cell.2007.12.034 18267076

[5] Venkiteswaran G., Lewellis S. W., Wang J., Reynolds E., Nicholson C., and Knaut H. (2013) Generation and dynamics of an endogenous, self-generated signaling gradient across a migrating tissue. Cell 155, 674–687 10.1016/j.cell.2013.09.046 24119842 PMC3842034

[6] Karin N., Wildbaum G., and Thelen M. (2016) Biased signaling pathways via CXCR3 control the development and function of CD4+ T cell subsets. J. Leukoc. Biol. 99, 857–862 10.1189/jlb.2MR0915-441R 26657511

[7] Cruz-Orengo L., Holman D. W., Dorsey D., Zhou L., Zhang P., Wright M., McCandless E. E., Patel J. R., Luker G. D., Littman D. R., Russell J. H., and Klein R. S. (2011) CXCR7 influences leukocyte entry into the CNS parenchyma by controlling abluminal CXCL12 abundance during autoimmunity. J. Exp. Med. 208, 327–339 10.1084/jem.20102010 21300915 PMC3039853

[8] Zohar Y., Wildbaum G., Novak R., Salzman A. L., Thelen M., Alon R., Barsheshet Y., Karp C. L., and Karin N. (2014) CXCL11-dependent induction of FOXP3-negative regulatory T cells suppresses autoimmune encephalomyelitis. J. Clin. Invest. 124, 2009–2022 10.1172/JCI71951 24713654 PMC4001543

[9] Kalatskaya I., Berchiche Y. A., Gravel S., Limberg B. J., Rosenbaum J. S., and Heveker N. (2009) AMD3100 is a CXCR7 ligand with allosteric agonist properties. Mol. Pharmacol. 75, 1240–1247 10.1124/mol.108.053389 19255243

[10] Gravel S., Malouf C., Boulais P. E., Berchiche Y. A., Oishi S., Fujii N., Leduc R., Sinnett D., and Heveker N. (2010) The peptidomimetic CXCR4 antagonist TC14012 recruits β-arrestin to CXCR7: roles of receptor domains. J. Biol. Chem. 285, 37939–37943 10.1074/jbc.C110.147470 20956518 PMC2992227

[11] Rajagopal S., Kim J., Ahn S., Craig S., Lam C. M., Gerard N. P., Gerard C., and Lefkowitz R. J. (2010) β-Arrestin- but not G protein-mediated signaling by the “decoy” receptor CXCR7. Proc. Natl. Acad. Sci. U.S.A. 107, 628–632 10.1073/pnas.0912852107 20018651 PMC2818968

[12] Defea K. (2008) β-Arrestins and heterotrimeric G-proteins: collaborators and competitors in signal transduction. Br. J. Pharmacol. 153, S298–S309 18037927 10.1038/sj.bjp.0707508 PMC2268080

[13] Berchiche Y. A., Gravel S., Pelletier M. E., St-Onge G., and Heveker N. (2011) Different effects of the different natural CC chemokine receptor 2b ligands on β-arrestin recruitment, Gα_i_ signaling, and receptor internalization. Mol. Pharmacol. 79, 488–498 10.1124/mol.110.068486 21088225

[14] Rajagopal S., Bassoni D. L., Campbell J. J., Gerard N. P., Gerard C., and Wehrman T. S. (2013) Biased agonism as a mechanism for differential signaling by chemokine receptors. J. Biol. Chem. 288, 35039–35048 10.1074/jbc.M113.479113 24145037 PMC3853256

[15] Corbisier J., Galès C., Huszagh A., Parmentier M., and Springael J. Y. (2015) Biased signaling at chemokine receptors. J. Biol. Chem. 290, 9542–9554 10.1074/jbc.M114.596098 25614627 PMC4392259

[16] Nobles K. N., Xiao K., Ahn S., Shukla A. K., Lam C. M., Rajagopal S., Strachan R. T., Huang T. Y., Bressler E. A., Hara M. R., Shenoy S. K., Gygi S. P., and Lefkowitz R. J. (2011) Distinct phosphorylation sites on the β2-adrenergic receptor establish a barcode that encodes differential functions of β-arrestin. Sci. Signal. 4, ra51 21868357 10.1126/scisignal.2001707 PMC3415961

[17] Thomsen A. R. B., Plouffe B., Cahill T. J. 3rd, Shukla A. K., Tarrasch J. T., Dosey A. M., Kahsai A. W., Strachan R. T., Pani B., Mahoney J. P., Huang L., Breton B., Heydenreich F. M., Sunahara R. K., Skiniotis G., et al (2016) GPCR-G protein-β-arrestin super-complex mediates sustained G protein signaling. Cell 166, 907–919 10.1016/j.cell.2016.07.004 27499021 PMC5418658

[18] Kumari P., Srivastava A., Banerjee R., Ghosh E., Gupta P., Ranjan R., Chen X., Gupta B., Gupta C., Jaiman D., and Shukla A. K. (2016) Functional competence of a partially engaged GPCR-β-arrestin complex. Nat. Commun. 7, 13416 10.1038/ncomms13416 27827372 PMC5105198

[19] Cahill T. J. 3rd, Thomsen A. R., Tarrasch J. T., Plouffe B., Nguyen A. H., Yang F., Huang L. Y., Kahsai A. W., Bassoni D. L., Gavino B. J., Lamerdin J. E., Triest S., Shukla A. K., Berger B., Little J., 4th, et al (2017) Distinct conformations of GPCR-β-arrestin complexes mediate desensitization, signaling, and endocytosis. Proc. Natl. Acad. Sci. U.S.A. 114, 2562–2567 10.1073/pnas.1701529114 28223524 PMC5347553

[20] Shenoy S. K., and Lefkowitz R. J. (2011) β-Arrestin-mediated receptor trafficking and signal transduction. Trends Pharmacol. Sci. 32, 521–533 10.1016/j.tips.2011.05.002 21680031 PMC3159699

[21] Birdsong W. T., Arttamangkul S., Clark M. J., Cheng K., Rice K. C., Traynor J. R., and Williams J. T. (2013) Increased agonist affinity at the μ-opioid receptor induced by prolonged agonist exposure. J. Neurosci. 33, 4118–4127 10.1523/JNEUROSCI.4187-12.2013 23447620 PMC3711766

[22] van Koppen C. J., and Jakobs K. H. (2004) Arrestin-independent internalization of G protein-coupled receptors. Mol. Pharmacol. 66, 365–367 10.1124/mol.104.003822 15322226

[23] Gesty-Palmer D., Chen M., Reiter E., Ahn S., Nelson C. D., Wang S., Eckhardt A. E., Cowan C. L., Spurney R. F., Luttrell L. M., and Lefkowitz R. J. (2006) Distinct β-arrestin- and G protein-dependent pathways for parathyroid hormone receptor-stimulated ERK1/2 activation. J. Biol. Chem. 281, 10856–10864 10.1074/jbc.M513380200 16492667

[24] Magalhaes A. C., Dunn H., and Ferguson S. S. (2012) Regulation of GPCR activity, trafficking and localization by GPCR-interacting proteins. Br. J. Pharmacol. 165, 1717–1736 10.1111/j.1476-5381.2011.01552.x 21699508 PMC3372825

[25] Chung K. Y., Day P. W., Vélez-Ruiz G., Sunahara R. K., and Kobilka B. K. (2013) Identification of GPCR-interacting cytosolic proteins using HDL particles and mass spectrometry-based proteomic approach. PLoS One 8, e54942 10.1371/journal.pone.0054942 23372797 PMC3556083

[26] Galliera E., Jala V. R., Trent J. O., Bonecchi R., Signorelli P., Lefkowitz R. J., Mantovani A., Locati M., and Haribabu B. (2004) β-Arrestin-dependent constitutive internalization of the human chemokine decoy receptor D6. J. Biol. Chem. 279, 25590–25597 10.1074/jbc.M400363200 15084596

[27] McCulloch C. V., Morrow V., Milasta S., Comerford I., Milligan G., Graham G. J., Isaacs N. W., and Nibbs R. J. (2008) Multiple roles for the C-terminal tail of the chemokine scavenger D6. J. Biol. Chem. 283, 7972–7982 10.1074/jbc.M710128200 18201974

[28] Borroni E. M., Cancellieri C., Vacchini A., Benureau Y., Lagane B., Bachelerie F., Arenzana-Seisdedos F., Mizuno K., Mantovani A., Bonecchi R., and Locati M. (2013) β-Arrestin-dependent activation of the cofilin pathway is required for the scavenging activity of the atypical chemokine receptor D6. Sci. Signal. 6, ra30.1–11, S1–3 23633677 10.1126/scisignal.2003627

[29] Benredjem B., Girard M., Rhainds D., St-Onge G., and Heveker N. (2017) Mutational analysis of atypical chemokine receptor 3 (ACKR3/CXCR7) interaction with its chemokine ligands CXCL11 and CXCL12. J. Biol. Chem. 292, 31–42 10.1074/jbc.M116.762252 27875312 PMC5217689

[30] Angel T. E., Chance M. R., and Palczewski K. (2009) Conserved waters mediate structural and functional activation of family A (rhodopsin-like) G protein-coupled receptors. Proc. Natl. Acad. Sci. U.S.A. 106, 8555–8560 10.1073/pnas.0903545106 19433801 PMC2688986

[31] Ballesteros J., and Weinstein H. (1995) Integrated methods for the construction of three-dimensional models and computational probing of structure-function relations in G-protein-coupled receptors. Methods Neurosci. 25, 366–428 10.1016/S1043-9471(05)80049-7

[32] Zhang W. B., Navenot J. M., Haribabu B., Tamamura H., Hiramatu K., Omagari A., Pei G., Manfredi J. P., Fujii N., Broach J. R., and Peiper S. C. (2002) A point mutation that confers constitutive activity to CXCR4 reveals that T140 is an inverse agonist and that AMD3100 and ALX40–4C are weak partial agonists. J. Biol. Chem. 277, 24515–24521 10.1074/jbc.M200889200 11923301

[33] Naumann U., Cameroni E., Pruenster M., Mahabaleshwar H., Raz E., Zerwes H. G., Rot A., and Thelen M. (2010) CXCR7 functions as a scavenger for CXCL12 and CXCL11. PLoS One 5, e9175 10.1371/journal.pone.0009175 20161793 PMC2820091

[34] Boularan C., Scott M. G., Bourougaa K., Bellal M., Esteve E., Thuret A., Benmerah A., Tramier M., Coppey-Moisan M., Labbé-Jullié C., Fåhraeus R., and Marullo S. (2007) β-Arrestin 2 oligomerization controls the Mdm2-dependent inhibition of p53. Proc. Natl. Acad. Sci. U.S.A. 104, 18061–18066 10.1073/pnas.0705550104 17984062 PMC2084296

[35] Eichel K., Jullié D., and von Zastrow M. (2016) β-Arrestin drives MAP kinase signalling from clathrin-coated structures after GPCR dissociation. Nat. Cell Biol. 18, 303–310 10.1038/ncb3307 26829388 PMC4767649

[36] Canals M., Scholten D. J., de Munnik S., Han M. K., Smit M. J., and Leurs R. (2012) Ubiquitination of CXCR7 controls receptor trafficking. PLoS One 7, e34192 10.1371/journal.pone.0034192 22457824 PMC3311620

[37] Venkatakrishnan A. J., Deupi X., Lebon G., Tate C. G., Schertler G. F., and Babu M. M. (2013) Molecular signatures of G-protein-coupled receptors. Nature 494, 185–194 10.1038/nature11896 23407534

[38] Warne T., Edwards P. C., Leslie A. G., and Tate C. G. (2012) Crystal structures of a stabilized β1-adrenoceptor bound to the biased agonists bucindolol and carvedilol. Structure 20, 841–849 10.1016/j.str.2012.03.014 22579251 PMC3384003

[39] Steen A., Larsen O., Thiele S., and Rosenkilde M. M. (2014) Biased and G protein-independent signaling of chemokine receptors. Front. Immunol. 5, 277 25002861 10.3389/fimmu.2014.00277 PMC4066200

[40] Katritch V., Fenalti G., Abola E. E., Roth B. L., Cherezov V., and Stevens R. C. (2014) Allosteric sodium in class A GPCR signaling. Trends Biochem. Sci. 39, 233–244 10.1016/j.tibs.2014.03.002 24767681 PMC4106411

[41] Gustavsson M., Wang L., van Gils N., Stephens B. S., Zhang P., Schall T. J., Yang S., Abagyan R., Chance M. R., Kufareva I., and Handel T. M. (2017) Structural basis of ligand interaction with atypical chemokine receptor 3. Nat. Commun. 8, 14135 10.1038/ncomms14135 28098154 PMC5253664

[42] Fenalti G., Giguere P. M., Katritch V., Huang X. P., Thompson A. A., Cherezov V., Roth B. L., and Stevens R. C. (2014) Molecular control of δ-opioid receptor signalling. Nature 506, 191–196 10.1038/nature12944 24413399 PMC3931418

[43] Cabana J., Holleran B., Leduc R., Escher E., Guillemette G., and Lavigne P. (2015) Identification of distinct conformations of the angiotensin-II type 1 receptor associated with the G_q/11_ protein pathway and the β-arrestin pathway using molecular dynamics simulations. J. Biol. Chem. 290, 15835–15854 10.1074/jbc.M114.627356 25934394 PMC4505491

[44] Rovati G. E., Capra V., and Neubig R. R. (2007) The highly conserved DRY motif of class A G protein-coupled receptors: beyond the ground state. Mol. Pharmacol. 71, 959–964 10.1124/mol.106.029470 17192495

[45] Rasmussen S. G., DeVree B. T., Zou Y., Kruse A. C., Chung K. Y., Kobilka T. S., Thian F. S., Chae P. S., Pardon E., Calinski D., Mathiesen J. M., Shah S. T., Lyons J. A., Caffrey M., Gellman S. H., et al (2011) Crystal structure of the β2 adrenergic receptor-G_s_ protein complex. Nature 477, 549–555 10.1038/nature10361 21772288 PMC3184188

[46] Deupi X., Edwards P., Singhal A., Nickle B., Oprian D., Schertler G., and Standfuss J. (2012) Stabilized G protein binding site in the structure of constitutively active metarhodopsin-II. Proc. Natl. Acad. Sci. U.S.A. 109, 119–124 10.1073/pnas.1114089108 22198838 PMC3252945

[47] Szczepek M., Beyrière F., Hofmann K. P., Elgeti M., Kazmin R., Rose A., Bartl F. J., von Stetten D., Heck M., Sommer M. E., Hildebrand P. W., and Scheerer P. (2014) Crystal structure of a common GPCR-binding interface for G protein and arrestin. Nat. Commun. 5, 4801 10.1038/ncomms5801 25205354 PMC4199108

[48] Kang Y., Zhou X. E., Gao X., He Y., Liu W., Ishchenko A., Barty A., White T. A., Yefanov O., Han G. W., Xu Q., de Waal P. W., Ke J., Tan M. H., Zhang C., et al (2015) Crystal structure of rhodopsin bound to arrestin by femtosecond X-ray laser. Nature 523, 561–567 10.1038/nature14656 26200343 PMC4521999

[49] Valentin-Hansen L., Frimurer T. M., Mokrosinski J., Holliday N. D., and Schwartz T. W. (2015) Biased G_s_ *versus* G_q_ proteins and β-arrestin signaling in the NK1 receptor determined by interactions in the water hydrogen bond network. J. Biol. Chem. 290, 24495–24508 10.1074/jbc.M115.641944 26269596 PMC4591830

[50] Bonde M. M., Hansen J. T., Sanni S. J., Haunsø S., Gammeltoft S., Lyngsø C., and Hansen J. L. (2010) Biased signaling of the angiotensin II type 1 receptor can be mediated through distinct mechanisms. PLoS One 5, e14135 10.1371/journal.pone.0014135 21152433 PMC2994726

[51] Bot G., Blake A. D., Li S., and Reisine T. (1998) Mutagenesis of the mouse δ opioid receptor converts (−)-buprenorphine from a partial agonist to an antagonist. J. Pharmacol. Exp. Ther. 284, 283–290 9435189

[52] Proulx C. D., Holleran B. J., Boucard A. A., Escher E., Guillemette G., and Leduc R. (2008) Mutational analysis of the conserved Asp2.50 and ERY motif reveals signaling bias of the urotensin II receptor. Mol. Pharmacol. 74, 552–561 10.1124/mol.108.045054 18509066

[53] Rahmeh R., Damian M., Cottet M., Orcel H., Mendre C., Durroux T., Sharma K. S., Durand G., Pucci B., Trinquet E., Zwier J. M., Deupi X., Bron P., Banères J. L., Mouillac B., and Granier S. (2012) Structural insights into biased G protein-coupled receptor signaling revealed by fluorescence spectroscopy. Proc. Natl. Acad. Sci. U.S.A. 109, 6733–6738 10.1073/pnas.1201093109 22493271 PMC3340029

[54] Wacker D., Wang C., Katritch V., Han G. W., Huang X. P., Vardy E., McCorvy J. D., Jiang Y., Chu M., Siu F. Y., Liu W., Xu H. E., Cherezov V., Roth B. L., and Stevens R. C. (2013) Structural features for functional selectivity at serotonin receptors. Science 340, 615–619 10.1126/science.1232808 23519215 PMC3644390

[55] Katritch V., Cherezov V., and Stevens R. C. (2013) Structure-function of the G protein-coupled receptor superfamily. Annu. Rev. Pharmacol. Toxicol. 53, 531–556 10.1146/annurev-pharmtox-032112-135923 23140243 PMC3540149

[56] Colvin R. A., Campanella G. S., Sun J., and Luster A. D. (2004) Intracellular domains of CXCR3 that mediate CXCL9, CXCL10, and CXCL11 function. J. Biol. Chem. 279, 30219–30227 10.1074/jbc.M403595200 15150261

[57] Vines C. M., Revankar C. M., Maestas D. C., LaRusch L. L., Cimino D. F., Kohout T. A., Lefkowitz R. J., and Prossnitz E. R. (2003) *N*-Formyl peptide receptors internalize but do not recycle in the absence of arrestins. J. Biol. Chem. 278, 41581–41584 10.1074/jbc.C300291200 12947104

[58] Mahabaleshwar H., Tarbashevich K., Nowak M., Brand M., and Raz E. (2012) β-Arrestin control of late endosomal sorting facilitates decoy receptor function and chemokine gradient formation. Development 139, 2897–2902 10.1242/dev.080408 22791893

[59] Luker K. E., Gupta M., Steele J. M., Foerster B. R., and Luker G. D. (2009) Imaging ligand-dependent activation of CXCR7. Neoplasia 11, 1022–1035 10.1593/neo.09724 19794961 PMC2745668

[60] Tian X., Irannejad R., Bowman S. L., Du Y., Puthenveedu M. A., von Zastrow M., and Benovic J. L. (2016) The α-arrestin ARRDC3 regulates the endosomal residence time and intracellular signaling of the β_2_-adrenergic receptor. J. Biol. Chem. 291, 14510–14525 10.1074/jbc.M116.716589 27226565 PMC4938174

[61] Weinman E. J., Hall R. A., Friedman P. A., Liu-Chen L. Y., and Shenolikar S. (2006) The association of NHERF adaptor proteins with G protein-coupled receptors and receptor tyrosine kinases. Annu. Rev. Physiol. 68, 491–505 10.1146/annurev.physiol.68.040104.131050 16460281

[62] Puthenveedu M. A., and von Zastrow M. (2006) Cargo regulates clathrin-coated pit dynamics. Cell 127, 113–124 10.1016/j.cell.2006.08.035 17018281

[63] Hoffmann F., Müller W., Schütz D., Penfold M. E., Wong Y. H., Schulz S., and Stumm R. (2012) Rapid uptake and degradation of CXCL12 depend on CXCR7 carboxyl-terminal serine/threonine residues. J. Biol. Chem. 287, 28362–28377 10.1074/jbc.M111.335679 22736769 PMC3436560

