# Supplementary material for: Ligand-specific conformational transitions and intracellular transport are required for atypical chemokine receptor 3–mediated chemokine scavenging
Source: J Biol Chem. 2017 Nov 27;293(3):893–905. doi: 10.1074/jbc.M117.814947 (PMC5777261; doi:10.1074/jbc.M117.814947)
Supplement: Supporting Information [file supp_293_3_893__index.html]

Ligand-specific conformational transitions and intracellular transport required for atypical chemokine receptor 3-mediated chemokine scavenging — Ligand-specific conformational transitions and intracellular transport are required for atypical chemokine receptor 3–mediated chemokine scavenging — ACKR3 conformations and intracellular transport — Supporting Information 

# Ligand-specific conformational transitions and intracellular transport are required for atypical chemokine receptor 3–mediated chemokine scavenging

## Supporting Information

- Supplemental data (.pdf, 899 KB) - Suppelemental data S1
