# Supplementary material for: Ligand-specific conformational transitions and intracellular transport are required for atypical chemokine receptor 3–mediated chemokine scavenging
Source: J Biol Chem. 2017 Nov 27;293(3):893–905. doi: 10.1074/jbc.M117.814947 (PMC5777261; doi:10.1074/jbc.M117.814947)

Supplemental Figure S1 shows arrestin relocalization at different time points after stimulation with 100nM of CXCL12 or CXCL11, as indicated.

Methods as indicated in the methods section.

Supplemental Figure S1

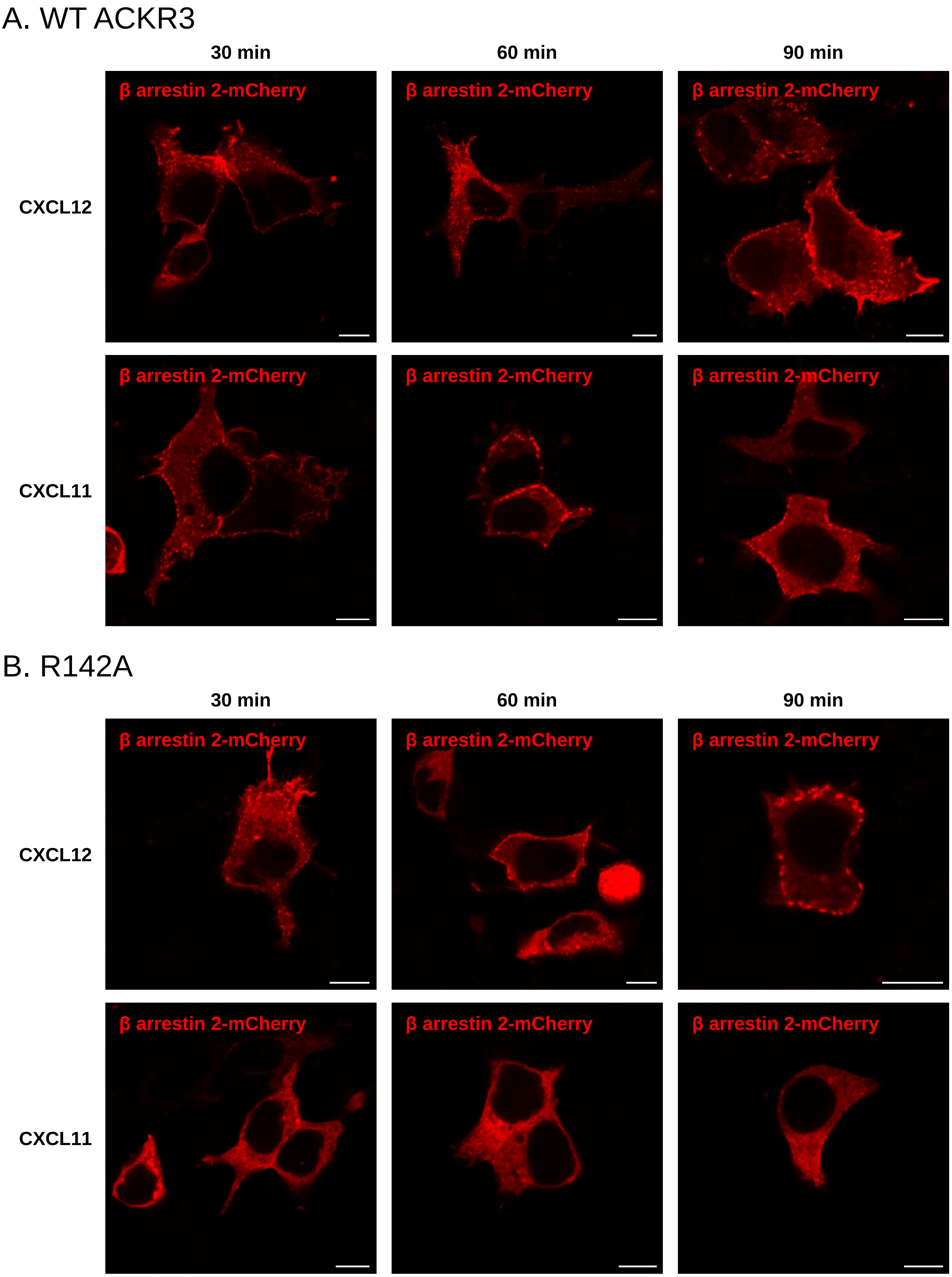

Supplement: Supporting Information [file 10.1074_M117.814947_jbc.M117.814947-1.pdf]
